# Disease-Modifying Effects of Lenvatinib, a Multiple Receptor Tyrosine Kinase Inhibitor, on Posttraumatic Osteoarthritis of the Knee

**DOI:** 10.3390/ijms25126514

**Published:** 2024-06-13

**Authors:** Yasuyuki Sogo, Eriko Toyoda, Toshihiro Nagai, Takumi Takahashi, Daichi Takizawa, Masahiko Watanabe, Masato Sato

**Affiliations:** 1 Department of Orthopaedic Surgery, Surgical Science, School of Medicine, Tokai University, 143 Shimokasuya, Isehara 259-1193, Kanagawa, Japan; sogosogo@tokai.ac.jp (Y.S.); toyoda.eriko.r@tokai.ac.jp (E.T.); takumi.takahashi22@gmail.com (T.T.); seikei-takizawa@tokai-u.jp (D.T.); masahiko@tokai.ac.jp (M.W.); 2 Center for Musculoskeletal innovative Research and Advancement (C-MiRA), Graduate School of Medicine, Tokai University, 143 Shimokasuya, Isehara 259-1193, Kanagawa, Japan; 3 Department of Orthopaedic Surgery, Tokai University Hachioji Hospital, 1838 Ishikawa-cho, Hachioji 192-0032, Kanagawa, Japan; nt6870@tokai.ac.jp

**Keywords:** osteoarthritis of the knee, lenvatinib, articular cartilage, synovitis, disease-modifying effects

## Abstract

Angiogenesis and vascular endothelial growth factor (VEGF) are involved in osteoarthritis (OA). We previously reported the inhibitory effect of bevacizumab in a rabbit model of OA. In the current study, we investigated the effects of lenvatinib, an angiogenesis inhibitor targeting the VEGF and fibroblast growth factor receptors, on synovitis, osteophyte formation, and cartilage degeneration in a rabbit OA model. Posttraumatic OA was induced by anterior cruciate ligament transection (ACLT) on one knee of each rabbit. Rabbits were placed into four groups according to the following lenvatinib doses: untreated control (*n* = 12), L0.3: 0.3 mg/kg/day (*n* = 15), L1.0: 1.0 mg/kg/day (*n* = 14), and L3.0: 3.0 mg/kg/day (*n* = 13) groups. We evaluated limb pain using the weight distribution ratio measured with an incapacitance tester, macroscopic osteophyte formation, and femoral condyle synovium and cartilage histology. For cartilage evaluation, the following distal sites of the femur were evaluated separately: femoral–tibial (FT), femoral–patellar (FP), and femoral corner (between FP and FT). The weight distribution ratio at 12 weeks after surgery was higher in the L0.3 and L1.0 groups than in the control group. Osteophyte formation and synovitis scores were significantly lower in the L0.3, L1.0, and L3.0 groups than in the control group. The Osteoarthritis Research Society International scores of the FT, corner, and FP sites in the L0.3 group were lower than in the control group. The cartilage thickness ratio at the FT and corner sites was significantly lower in the L0.3 group than in the control group. Krenn’s grading system of cartilage synovitis showed that all lenvatinib-administered groups had significantly lower scores than the control group. MMP3 expression level in cartilage tissue was significantly lower in the L3.0 group compared with the other three groups. ADAMTS5 expression was lower in the L3.0 group compared with the control and L0.3 groups. Oral administration of lenvatinib inhibited synovitis, osteophyte formation, and cartilage degeneration and reduced pain in a rabbit ACLT model. Lenvatinib is an oral VEGF inhibitor that is easier to administer than other VEGF inhibitors and may have potential as a treatment of posttraumatic OA.

## 1. Introduction

Osteoarthritis (OA) is increasing in prevalence because of longer life expectancy and an increasing population of older people. It is estimated that, in 2020, nearly 60 million Americans experienced some kind of OA [1]. A degenerative disease of the joints, OA is characterized by the destruction of the articular cartilage, inflammation of the synovial membrane, meniscal degeneration, inflammation and fibrosis of the infrapatellar fat pad, and bone spur formation. As the disease progresses, activities such as walking become difficult because of pain in the joints, and this impairment can interfere with daily life [2].

Although several risk factors are associated with OA, including genetic predisposition [3], aging [4], obesity [5], trauma [6], gender [7], and joint malalignment [8], it is unknown which of these pathogenetic processes occurs first. The treatment of OA is multidisciplinary and involves physical therapy, medication, intra-articular injection, and surgery [9]. For patients with progressive OA, the therapeutic effects of conservative therapy are limited, and surgery is often required. Pain stimulation and increased inflammatory cytokine production promote angiogenesis by releasing growth factors from cells such as macrophages [10]. Unlike vascular-rich bone tissue, cartilage tissue is an avascular tissue with limited regenerative ability. Angiogenesis is induced in osteochondral areas and the synovium, leading to calcification of articular cartilage and synovitis [11] accompanying vascularization of infrapatellar fat pad [12,13], which contribute to further stimulation of angiogenesis and inflammatory changes that lead to OA [14].

Although much research has focused on elucidating the pathophysiological mechanisms of OA, there are no established drugs for clinical use to prevent or slow the progression of this disease [9]. Among the pathological mechanisms, angiogenesis plays an important role in the initiation and progression of OA and is a potential therapeutic target for OA. In the later stages of OA, invasion of blood vessels from the subchondral bone and synovitis are induced by angiogenesis, and osteophyte growth occurs. Vascular endothelial growth factor (VEGF) has been reported to play an important role in the development of OA. Studies have revealed a central role for VEGF in synovitis and osteophyte formation in OA pathogenesis [15]. Nagai et al. reported that inhibition of VEGF signaling had an OA-suppressive effect and a positive effect on cartilage regeneration [16]. In the study, bevacizumab, an anti-VEGF monoclonal antibody, was administered intravenously or intra-articularly in an osteochondral defect model and was found to reduce synovitis, osteophyte formation, and cartilage degeneration. The authors suggested that the intra-articular administration of bevacizumab could reduce the risk of adverse events compared with intravenous administration [17]. We hypothesized that inhibition of VEGFR signaling prevents OA progression.

Lenvatinib is an oral multikinase inhibitor that selectively inhibits VEGFRs 1 to 3 and other proangiogenic and pro-oncogenic receptor tyrosine kinases, including FGFRs 1 to 4, platelet-derived growth factor receptor α, KIT, and RET [18]. Lenvatinib is effective and safe for treating multiple cancer types [19]. Inhibition of VEGF signaling increased the production of FGF-2 and compensatively led to increased FGFR signaling and reactivation of angiogenesis in a mouse model of pancreatic islet carcinogenesis [20]. Thus, the inhibitory effect on FGFR1-4 may be advantageous if compensative FGFR-dependent angiogenesis is involved in OA pathogenesis. Meanwhile, there is a possibility that joint homeostasis is disturbed via FGF signal inhibition.

To assess the potential of lenvatinib as an oral drug for traumatic OA, we investigated its effects on synovitis, osteophyte formation, and cartilage degradation in a rabbit OA model induced by anterior cruciate ligament transection (ACLT).

## 2. Results

### 2.1. Preliminary Experiment

**Figure 1:**
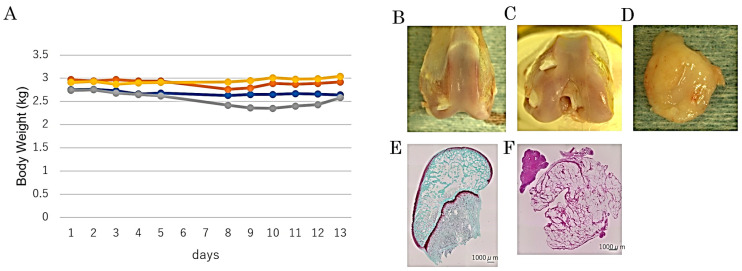
Assessment of tolerability of lenvatinib administration on rabbits. (**A**) Effect on body weight gain of rabbits treated with 3.0 mg/kg of lenvatinib for 5 days in preliminary experiment. (**B**–**F**) Typical macroscopic and histological findings for the cartilage and synovium 13 days after administration of lenvatinib in the preliminary experiment. (**B**,**C**) Macroscopic view of femoral condyle. No osteophyte formation was observed. (**D**) Macroscopic view of synovial tissue. (**E**) Sagittal section of femur showed no evidence of decreased Safranin O staining and erosion. (**F**) HE-stained section of synovial tissue. (**B**,**C**) No osteophyte formation was observed in the femoral condyles, and HE-stained images of the cartilage tissue showed no evidence of decreased Safranin O staining and erosion.

We evaluated adverse events in these rabbits after lenvatinib administration. We administered 3.0 mg/kg per day of lenvatinib to normal rabbits (*n* = 4) for 5 days and measured body weight gain and food consumption for 14 days after administration (Figure 1A). None of the group of rabbits showed decreased dietary intake or weight gain. Fourteen days after administration of lenvatinib, no osteophyte formation was observed in the femoral condyles, and images of the cartilage tissue sections showed no evidence of decreased Safranin O staining or erosion (Figure 1B–F). We concluded that a 3.0 mg/kg dosage can be safely administered to rabbits without serious adverse events on joint homeostasis.

### 2.2. Application of Lenvatinib on ACLT-Induced Osteoarthritis Model in Rabbit

#### 2.2.1. Adverse Events

**Figure 2:**
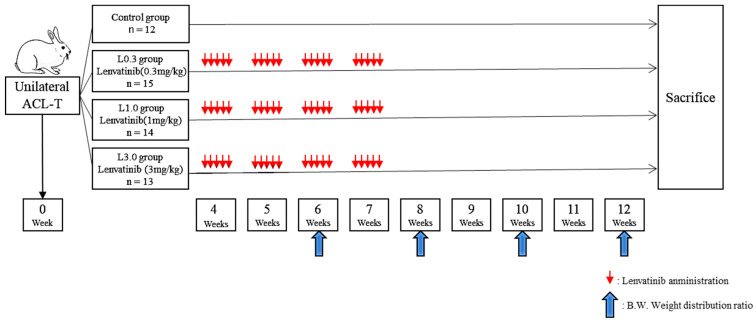
Experimental design. ACLT was performed on one knee of 54 rabbits to induce posttraumatic OA and pain behavior was assessed by measuring weight-bearing asymmetry. Lenvatinib was administered orally 5 days per week from 4 to 7 weeks after ACLT. We classified the rabbits into four groups according to the following lenvatinib dosages: The control group was untreated (*n* = 12). The lenvatinib groups were the L0.3 group (0.3 mg/kg/day, *n* = 15), the L1.0 group (1.0 mg/kg/day, *n* = 14), and the L3.0 group (3.0 mg/kg/day, *n* = 13). All rabbits were sacrificed 12 weeks after surgery, and the histology and gene expression were analyzed. As an indicator of pain, we measured the weight distribution ratio of the damaged versus undamaged limb using a Linton incapacitance tester at 6, 8, 10, and 12 weeks after ACLT. Rabbits were sacrificed after the experiments by an overdose of intravenous anesthetic at 12 weeks after ACLT.

**Figure 3:**
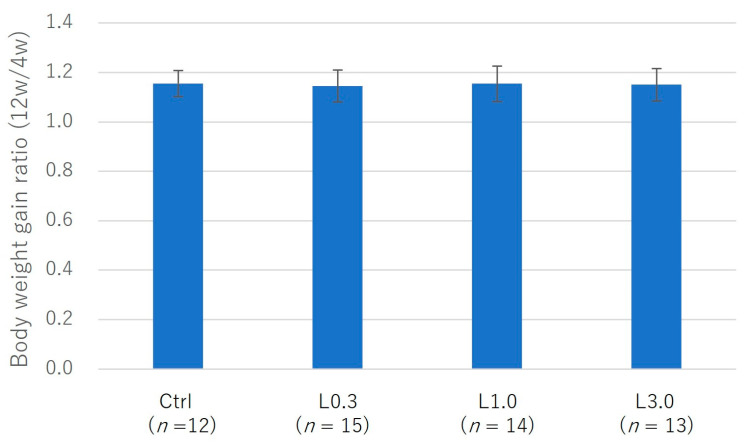
Body weight changes in rabbits and the ratio of weight gain from before to after lenvatinib administration in ACLT rabbits. Dietary intake did not decrease, and no digestive symptoms, such as diarrhea, were observed in any group. Body weight gain and the weight gain ratio did not differ significantly between the groups before and after lenvatinib administration.

The experimental design is depicted in Figure 2. We observed no decrease in dietary intake or indicators of digestive symptoms, such as diarrhea, in any of the four groups. We measured body weight before lenvatinib administration (4 weeks after ACLT) and after lenvatinib administration (12 weeks after ACLT) and calculated the body weight gain ratio. There was a trend toward less weight gain in the L3.0 group, but this was because of differences at the start of treatment. Body weight and the weight gain ratio did not differ significantly between the four groups before and after lenvatinib administration (Figure 3).

#### 2.2.2. Pain Evaluation

**Figure 4:**
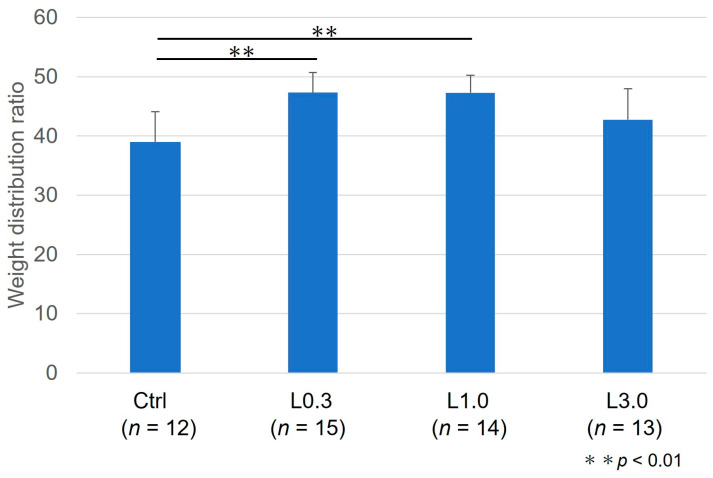
Weight distribution ratio of the damaged to undamaged limb 12 weeks after ACLT. The limb weight distribution ratio was higher in the L0.3 and L1.0 groups than in group A. Data represent mean ± SD ** *p* < 0.01. Tukey’s HSD post hoc test was used.

Twelve weeks after ACLT, we measured the weight distribution ratio between hind limbs. The damaged limb weight distribution ratio was greater in the L0.3 and L1.0 groups than in the control group (Figure 4).

#### 2.2.3. Comparison of Osteophyte Formation

**Figure 5:**
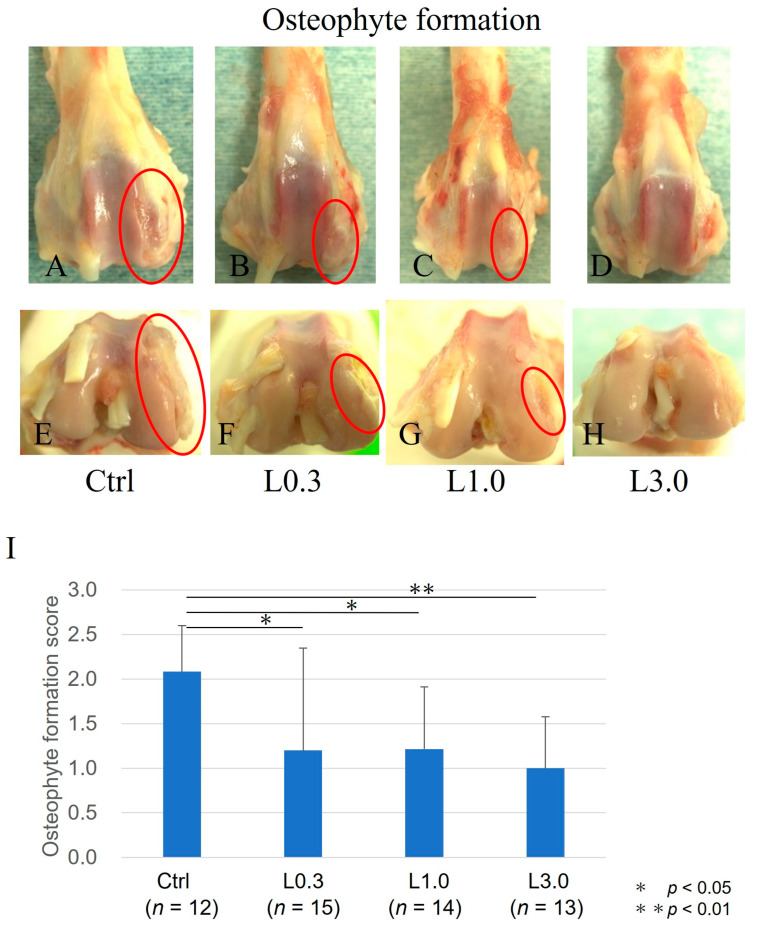
Osteophyte formation at 12 weeks after ACLT. Osteophyte formation was evaluated macroscopically in the joints from each group 12 weeks after ACLT. (**A**–**H**) Representative images of the articular cartilage surface. (**A**–**D**) Images of the patellar surface of the femur. (**E**–**H**) Images of the condyle of the femur. (**I**) Morphology of articular cartilage was evaluated macroscopically using an osteophyte formation score developed by Tibesku et al. [21]. The criteria for macroscopic grading were as follows: grade 0 (absent), grade 1 (mild osteophyte formation), grade 2 (moderate osteophyte formation), and grade 3 (severe osteophyte formation). Osteophyte formation was observed in the control group but was suppressed, and the articular cartilage surface was smooth in the L0.3, L1.0, and L3.0 groups. The osteophyte formation score was lower in the three lenvatinib groups and lowest in the L0.3 group. Data are expressed as the mean ± SD. Tukey’s HSD post hoc test was used. Differences were considered significant for *p*-values < 0.05.

Twelve weeks after ACLT, macroscopic evaluation of joints identified osteophyte formation. Osteophyte formation was observed in the control group, but less formation was observed, and the articular cartilage surface was smooth in the L0.3, L1.0, and L3.0 groups. The osteophyte formation score was significantly lower in all lenvatinib groups than in the control group and was lowest in the L3.0 group (Figure 5).

#### 2.2.4. Histological Evaluation of the Articular Cartilage

**Figure 6:**
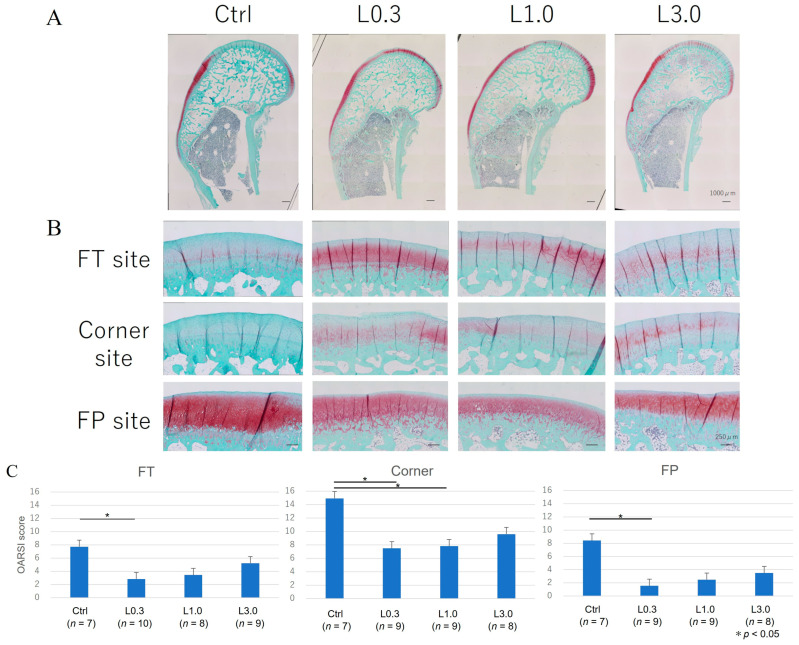
Histological evaluation of cartilage using the OARSI score. (**A**) Representative images of the sagittal section of the femur. Each section was stained with Safranin O. (**B**) Magnified images for the divided area for evaluation; the medial femoral condyle at the femoral–tibial (FT), femoral–patellar (FP), and corner sites in the articular cartilage area. Sections showed greater Safranin O-positive staining in the lenvatinib groups and less staining in the control group. The articular cartilage in the control group showed delamination of the superficial layer and erosion of hyaline cartilage. (**C**) The evaluations of OA repair sites at the medial femoral condyle were conducted semi-quantitatively using the OARSI modified Mankin score grading and staging system. The total OARSI score was significantly lower in the FT, corner, and FP sites in the L0.3 group than in the control. Data are expressed as the mean ± SD. Tukey’s HSD post hoc test was used. Differences were considered significant for *p*-values < 0.05.

We evaluated the medial femoral condyle at the articular cartilage area’s FT, FP, and corner sites (Figure 6). The histological assessment showed greater Safranin O-positive staining in the lenvatinib administration groups than in the control group. The articular cartilage in the control group showed delamination of the superficial layer and erosion of hyaline cartilage. The total OARSI score was significantly lower at the FT, FP, and corner sites in the L0.3 group than in the control group [22].

**Figure 7:**
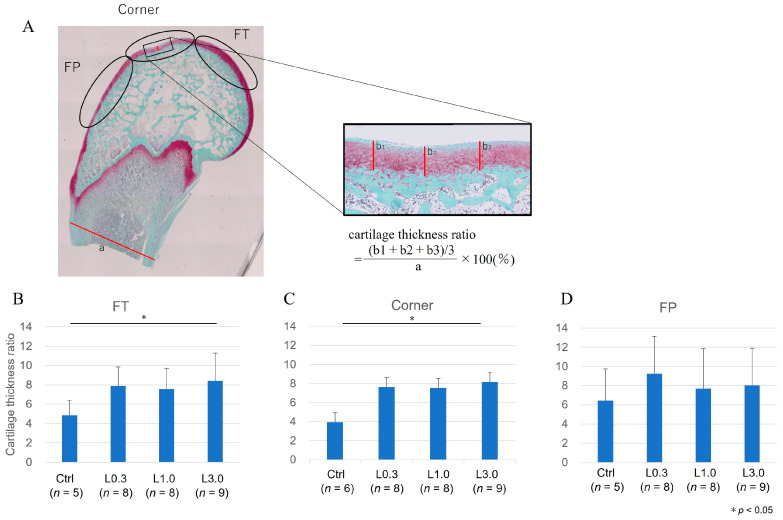
Comparison of cartilage tissue thickness ratios between groups. (**A**) Calculation of cartilage thickness ratios. The thickness of the cartilage was taken as the distance from the cartilage surface to the subchondral bone. The ratio of cartilage thickness in each part of the femur was calculated relative to the bone axis measured distal to the growth plate. (**B**–**D**) The cartilage thickness ratio for each area: the medial femoral condyle at the FT, femoral–patellar (FP), and corner sites. The ratio was lower in the L0.3 and L1.0 groups than in the control group for the corner site and in the L0.3 than in the control group for the FT site. Data are expressed as the mean ± SD. Tukey’s HSD post hoc test was used. Differences were considered significant for *p*-values < 0.05.

We examined the repair of cartilage thickness, which is expressed as the cartilage thickness ratio. The cartilage thickness ratio was lower in the L0.3 and L1.0 groups than in the control group for the corner site and lower in the L0.3 than in the control group for the FT site (Figure 7).

#### 2.2.5. Histological Evaluation of the Synovium

**Figure 8:**
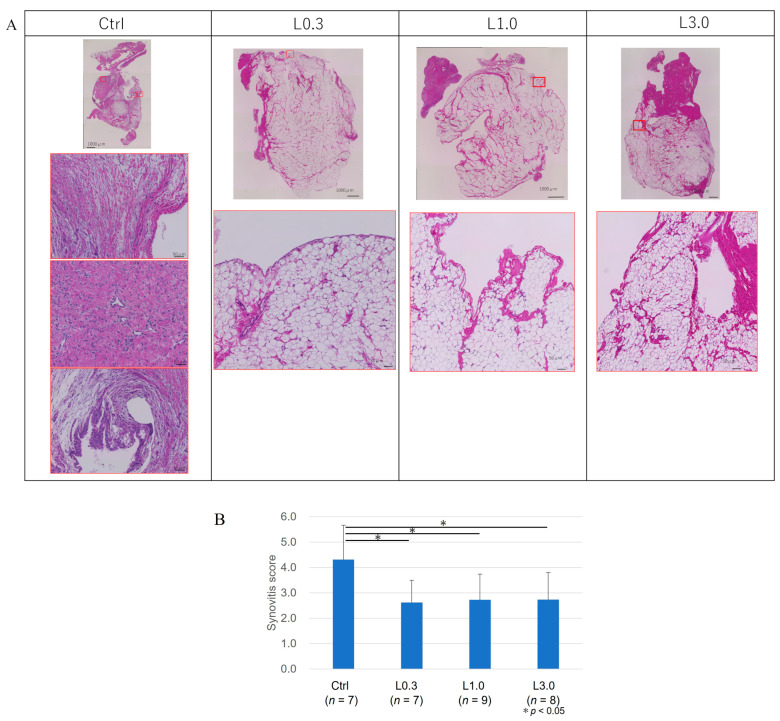
Krenn’s histological evaluation of the synovium 12 weeks after ACLT. (**A**) Representative images of synovium. Each section was stained with H&E. The control samples exhibited strong indications of synovitis and had a higher synovitis score. In the control group, the synovial lining layer was enlarged, large giant cells and lymphocytes were frequent, and the synovial stroma exhibited high cellularity with densely located fibroblast-like cells and cytological signs of activation. The synovial cells were accompanied by lymphoid follicles and very dense lymphocyte and plasma cells, indicative of inflammatory infiltration. By contrast, in the lenvatinib-treated groups, synovitis was suppressed, and the synovitis score was lower. (**B**) We evaluated the extent of synovitis using Krenn’s evaluation scoring according to the three synovial membrane features: synovial lining cell layer, stroma cell density, and inflammatory infiltrate. The changes were scored as none (0), slight (1), moderate (2), and strong (3). The values for the synovial membrane features were summed and interpreted as 0–1, no synovitis; 2–4, low-grade synovitis; and 5–9, high-grade synovitis. Data are expressed as the mean ± SD. Tukey’s HSD post hoc test was used. Differences were considered significant for *p*-values < 0.05.

Twelve weeks after ACLT, the macroscopic appearance of the synovium showed enlargement with redness and recognized thick vascular invasion in the control group. By contrast, in the lenvatinib groups, the synovium was preserved and appeared pale yellow, although a thin vascular invasion was apparent. We evaluated the suppressive effect of synovitis according to Krenn’s evaluation. In the control group, the synovial lining cell layer was enlarged, the density of the synovial stroma was increased, and lymphocytic inflammatory infiltrate was observed [23]. Conversely, in lenvatinib groups, these findings of synovitis were suppressed, and the synovitis score also improved (Figure 8). We considered that the synovial membrane and infrapatellar fat pad act as an anatomo-functional unit, and these tissues were not distinguished [24].

#### 2.2.6. Gene Expression in Articular Cartilage

**Figure 9:**
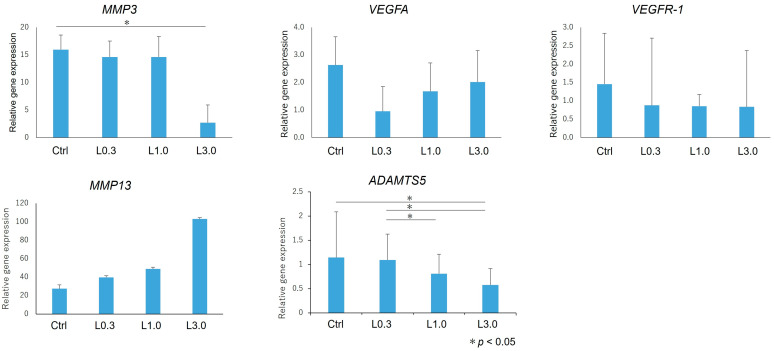
Gene expression in cartilage. We used real-time PCR to assess changes in the expression of genes encoding anabolic and catabolic factors, expressed relative to the levels in normal tissues at the baseline. *MMP3* and *ADAMTS5* expression levels were significantly lower in the L3.0 group than in the control group. *ADAMTS5* expression was also lower in the L3.0 group than in the L0.3 and L1.0 groups. Expression of other genes did not differ between groups. The relative expression of the target mRNA was standardized to glyceraldehyde phosphate dehydrogenase, and the expression level was calculated using the 2 −ΔΔCT values of the normal tissue. Data are expressed as the mean ± SD. Tukey’s HSD post hoc test was used. Differences were considered significant for *p*-values < 0.05.

We harvested the articular cartilage from each group 12 weeks after ACLT. We used real-time PCR to assess changes in the expression of genes involved in anabolic and catabolic factors, and we compared these changes in each group relative to the expression levels of the normal tissues at the baseline (Figure 9). *MMP3* expression was significantly lower in the L3.0 group compared with the control group. *ADAMTS5* expression was also decreased in the L3.0 group compared with the control group, and the L3.0 group also had decreased expression compared with L0.3 and L1.0 groups. There were no differences in gene expression in each group for *VEGFA*, *VEGFR-1*, and *MMP13*.

## 3. Discussion

During the later stages of OA in affected patients, *VEGF* expression is increased in the articular cartilage [25,26], synovium [27,28], synovial fluid [29], subchondral bone [30], and serum [15,31]. VEGF inhibits the anabolic function of chondrocytes. Decreased aggrecan and type II collagen expression have been reported in rat articular chondrocytes cultured in a VEGF medium [32]. Inhibition of VEGF in chondrocytes of human OA patients inhibits cartilage catabolism [33]. These studies suggest that VEGF may be involved in the development of OA and that VEGF inhibition may limit cartilage degeneration. Both MMP3 and MMP13 are matrix metalloproteinases that degrade collagen, but they have different substrate specificity and different modes of degradation of cartilage matrix. It is not surprising that the regulation of their expression in OAK is opposite. For the alteration of VEGF, some feedback signal induced by VEGFR or FGFR signal blockage might be involved. In addition, it has been reported that the catabolic and anabolic effects of FGF vary depending on the species [15,31]. The expression of FGF needs to be examined; we have mentioned this in the limitation section.

The intravenous or intra-articular administration of bevacizumab demonstrated articular cartilage repair in an osteochondral defect model [16,17]. In those studies, bevacizumab reduced synovitis, osteophyte formation, and cartilage degeneration. The results indicate that VEGF signaling is a potential target for OA treatment.

However, there are several obstacles to the application of bevacizumab in OA treatment. In addition to the relatively higher cost of treatments, the adverse effects of bevacizumab, such as adverse cardiovascular events, thromboembolic events, and increasing hemorrhage, are not tolerable for OA, which is not a life-threatening chronic disease. In addition, bevacizumab is an essential regimen for cancer treatment; the priority is to avoid the development of neutralizing antibodies for bevacizumab.

This study administered lenvatinib, a small-molecule VEGF inhibitor, orally. The dose of lenvatinib was equivalent to that used in human clinical practice, and no adverse events, such as weight loss, were observed in the rabbits in a preliminary experiment. Improvements in pain, suppression of synovitis and osteophyte formation, and cartilage repair were observed in the lenvatinib-treated groups. These results suggest the potential of lenvatinib as an orally available disease-modifying drug for OA.

Lenvatinib selectively inhibits VEGFRs 1–3 and other proangiogenic and pro-oncogenic receptor tyrosine kinases, including FGFRs 1–4, platelet-derived growth factor receptor, KIT, and RET [18]. Nagao et al. reported that anti-VEGF antibodies and oral administration of the VEGFR2 kinase inhibitor vandetanib suppress OA progression via inhibiting VEGFR2 signaling; meanwhile, inhibition of the VEGFR1 signal in dorsal root ganglia was associated with a reduction in pain [34]. The superior effects of pazopanib, which inhibits VEGFR1 and VEGFR2, compared with the VEGF2R-selective inhibitor vandetanib on a DMM mouse model were reported [35]. Lenvatinib can inhibit VEGFR1 and VEGFR2, suggesting a dual mechanism for OA symptoms, suppressing pain via VEGFR2 and cartilage protection and reducing synovitis via VEGFR1.

The inhibition of VEGF alone has been reported to reactivate angiogenesis through a compensatory increase in FGF [20,36]. Lenvatinib inhibits both the VEGFR and FGFR and is expected to have a greater inhibitory effect on angiogenesis than VEGFR inhibition alone. Lenvatinib inhibits angiogenesis in the synovial membrane, thereby reducing synovitis. FGF has different effects on cartilage metabolism in different species [37]. It has been reported that FGFR1 acts catabolically in humans [38], and lenvatinib is expected to be involved in cartilage repair through its anti-FGF effects. By contrast, FGFR3 has also been reported to have chondroprotective and anabolic effects [39], and future evaluations of the effects of FGF on cartilage degeneration and damage are needed.

The administration of lenvatinib in this rabbit model of OA suggests the possibility of tissue repair and regeneration and a significant osteophyte-suppression effect. However, although the osteophyte suppression and conserved cartilage thickness were greater in the L3.0 group, the overall cartilage tissue repair and regeneration, as measured by the OARSI score, was significant only in the L0.3 group. Lenvatinib has a superior inhibitory effect on VEGFR2 with an IC50 of 3.0 nmol/L than on VEGFR1 with 4.7 nmol/L [40]. It has been reported that VEGFR2 is associated with an OA-suppressing effect and VEGFR1 has a pain-suppressing effect [27]. Thus, there is a possibility that a lower dose caused only an OA-suppressing effect and a higher dose had effects of both OA suppression and pain suppression. The mechanisms by which lenvatinib affects tissue repair and regeneration should be studied in greater detail in future studies.

Studies of the effects of anti-VEGF agents in animal models of OA have reported effects on various tissues, including articular cartilage, subchondral bone, and synovial tissue [17,41]. The present study showed similar effects, such as cartilage degeneration and synovitis inhibition. Although anti-VEGF alone has potential in the treatment of posttraumatic OA, synergistic effects may occur when combined with other biological therapies. Intra-articular injection of platelet-rich plasma (PRP) is used widely in the treatment of OA. Because PRP contains several growth factors, including VEGF, blockade of VEGF function may augment the efficacy of PRP therapy. We speculate that combining PRP with anti-VEGF agents may have the potential to treat OA. In a rat model of OA, PRP given with anti-VEGF was reported to improve articular cartilage repair [42].

### Limitations

In this study, we evaluated the effects of lenvatinib on pain, the histology of synovial and cartilage tissues, and the expression of genes encoding catabolic factors in cartilage tissues; however, we did not verify gene expression in synovial tissues. The expression of *VEGF* and *VEGFR* in synovial tissues may have differed between the lenvatinib and control groups because lenvatinib is expected to suppress angiogenesis by inhibiting VEGFR. Compared with bevacizumab, which has only an anti-VEGF effect, lenvatinib also has an anti-FGFR effect, and the extent to which lenvatinib affects the synovial membrane and cartilage metabolism should be examined in the future. The administration period was 4 weeks in this study, and whether adverse events, such as weight loss and side effects in the joints, occur with longer administration is unknown. The dose–response relationship for lenvatinib was examined using three dosage groups. Since the effective dose differed between the various end-points examined, our results should be confirmed, and further work is needed to determine the optimal dose for controlling OA and relieving pain. Lenvatinib is an orally administered drug, but its efficacy in inhibiting OA by intravenous or intra-articular administration and oral administration should be considered. In this study, the synovial membrane and infrapatellar fat pad were histologically evaluated as an anatomo-functional unit. However, it is possible that differences in histological changes and gene expression may be observed in each tissue, and further study is needed.

## 4. Materials and Methods

### 4.1. Animals and Surgical Procedures

All procedures using animals in this study were performed in accordance with the *UK* Animals (Scientific Procedures) Act, 1986, the European Communities Council Directive of 24 November 1986 (86/609/EEC), the National Institutes of Health guide for the care and use of laboratory animals (NIH Publications No. 8023, revised 1978), and the ARRIVE statement [43]. Animal experiments were approved by the ethics review board and performed per the guidelines on animal use of Tokai University (Authorization Number 151084). Adolescent Japanese white rabbits (Tokyo Laboratory Animals Science Co., Ltd., Tokyo, Japan), aged 16 to 18 weeks and weighing about 2.5 kg, were used in this study. The rabbits were anesthetized by exposure to sevoflurane and O_2_ gas. Under sterile conditions, a medial parapatellar approach was employed to release the joint capsule and to perform the ACLT [17,44]. After recovery from surgery, all animals were allowed to walk freely in their cages without any splints.

### 4.2. Lenvatinib Dose

The half-life of lenvatinib in normal blood circulation is reportedly 17.8–34.5 days [45]. In humans, the approved dose of lenvatinib is 24 mg/kg, with a clinical administration interval of 24 h [46,47]. This dose corresponds to 1 mg/kg body surface area in rabbits. With reference to previous pharmacokinetic experiments involving lenvatinib and our preliminary tolerability experiment, we investigated lenvatinib at doses of 0.3 mg/kg, 1.0 mg/kg, and 3.0 mg/kg per day for 4 weeks.

### 4.3. Oral Administration of Lenvatinib

Lenvatinib (lenvatinib mesylate; CAS No. 417716-92-8; kindly provided by Eisai co. ltd., Tokyo, Japan) for use in animal experiments was dissolved in 3 mM hydrochloric acid [48,49], and the concentration was adjusted so that the dose was contained within 5 mL for each group. Stock solutions were stored at 4 *°C*. We anesthetized rabbits and administered lenvatinib to the rabbits through an orogastric tube administered orally 5 days weekly from week 4 to week 7 after ACLT. The dose of the stock solution was diluted with distilled water to the required volume (5 mL) and administered according to the rabbit’s body weight.

### 4.4. Experimental Design

To determine the treatment dose, the tolerable dosage of lenvatinib for rabbits was confirmed by the preliminary experiment. We administered lenvatinib at 3.0 mg/kg to normal rabbits for 5 days and measured body weight gain and food consumption for 13 days afterward (Figure 2).

To induce posttraumatic OA, ACLT was performed on one knee of each of 54 rabbits, and pain behavior was measured by assessing weight-bearing asymmetry. Lenvatinib was administered orally 5 days weekly from week 4 to week 7 after ACLT. Four groups of rabbits were analyzed according to the following lenvatinib dosages: The untreated control group (Ctrl) received no drug (*n* = 12). The lenvatinib-treated groups were designated as L0.3 (0.3 mg/kg/day; *n* = 15), L1.0 (1.0 mg/kg/day; *n* = 14), and L3.0 (3.0 mg/kg/day; *n* = 13). We measured the weight distribution ratio of the damaged to undamaged limb using the Linton incapacitance tester (Linton Instrumentation, Norfolk, UK) for pain evaluation 6, 8, 10, and 12 weeks after ACLT. All rabbits were sacrificed using an overdose of intravenous anesthetic 12 weeks after surgery, and the histology and gene expression in the joint tissue were analyzed.

### 4.5. Morphology of Osteophyte Formation

The femoral condyles were examined macroscopically. Two independent blinded observers evaluated the morphology of osteophyte formation using an osteophyte formation score developed by Tibesku et al. [20]. The criteria for macroscopic grading were as follows: grade 0 (absent), grade 1 (mild osteophyte formation), grade 2 (moderate osteophyte formation), and grade 3 (severe osteophyte formation) [20]. We included both osteophytes and chondro-osteophytes in the evaluation of osteophyte formation because both are regarded as neoplastic tissue caused by endochondral ossification resulting from angiogenesis in the articular margin associated with OA [49]. The formation of chondro-osteophytes by hypertrophic chondrocytes reflects the process of endochondral ossification in the growth of osteophytes. After morphological grading, the condyles were prepared for histological evaluation and gene expression analysis.

### 4.6. Histological Examination

Histology sections of the synovium and cartilage were stained for hematoxylin and eosin (H&E) and evaluated using a synovitis score developed by Krenn et al. [23].

The distal parts of the femur were excised and fixed in 4% paraformaldehyde for 7 days. Each specimen was decalcified in a 10% ethylenediaminetetraacetic acid solution in distilled water (pH 7.4) for 2 to 3 weeks, embedded in paraffin wax, and cut along the sagittal plane. Each section was stained with Safranin O. We divided the distal portion of the femur into the femoral–tibial (FT) site, femoral–patellar (FP) site, and corner site, which was between FP and FT.

We evaluated OA repair sites at the medial femoral condyle semi-quantitatively using the Osteoarthritis Research Society International (OARSI) modified Mankin score grading and staging system [21]. This system includes six histological grades and four histological stages. The total score (grade score multiplied by stage score) ranges from 1 point (normal articular cartilage) to 24 points (no repair). We also evaluated articular cartilage thickness as an indicator of cartilage repair. The thickness of the cartilage was taken as the distance from the cartilage surface to the subchondral bone. The ratio of cartilage thickness in each part of the femur was calculated relative to the bone axis measured distal to the growth plate. To minimize observer bias, two blinded observers examined the sections, and the scores were averaged.

The synovium was harvested from the infrapatellar fat pad region. The synovial membrane was fixed in 4% paraformaldehyde for 7 days and then embedded in paraffin. Each section was stained with H&E. We evaluated the extent of synovitis using Krenn’s evaluation scoring according to three synovial membrane features: synovial lining cell layer, stroma cell density, and inflammatory infiltrate [23]. The changes were scored as none (0), slight (1), moderate (2), and strong (3). The values for the synovial membrane features were summed and interpreted as 0–1, no synovitis; 2–4, low-grade synovitis; and 5–9, high-grade synovitis.

### 4.7. Pain Evaluation

A Linton incapacitance test meter was used to measure the weight distribution ratio as an indicator of pain, and the values were compared between the damaged and undamaged limbs. The measurements were obtained from rabbits after they were transferred into the rabbit holder. The weight distribution of both hind legs was measured ten times, and the following formula was used to calculate the damaged limb weight distribution ratio (%) obtained by loading the left and right limbs [10,11]:

Damaged limb weight distribution ratio (%) = {damaged limb load (g)/undamaged limb load (g) + damaged limb load (g)} × 100.

### 4.8. Gene Expression Analysis by Real-Time Polymerase Chain Reaction (PCR)

The articular cartilage was obtained from the femoral condyle. Tissue samples were homogenized in liquid nitrogen using a Cryo-Press (Microtec Nition, Chiba, Japan). Total RNA was isolated using the SV Total RNA Isolation System (Promega Corp., Madison, WI, USA), following the manufacturer’s instructions. RNA quantification and quality were determined using the 260/280 nm ratio. Each RNA sample was then reverse-transcribed to cDNA using TaqMan Reverse Transcription reagents (Applied Biosystems, Foster City, CA, USA) in a thermocycler set at 42 °C for 60 min and at 95 °C for 5 min. The primer sequences used in this study are listed in Table 1. Real-time PCR was performed in an ABI SDS 7300 real-time PCR system (Applied Biosystems) using SYBR Green Master Mix (Applied Biosystems). cDNA (2 μL) was added to bring the final volume of the real-time PCR sample to 25 μL. We then ran 35 to 45 amplification cycles of 15 s at 95 °C, 1 min at 60 °C. Normal articular cartilage was used as a reference for gene expression comparisons. The target mRNA was standardized to glyceraldehyde phosphate dehydrogenase, and the expression level was calculated using the 2^−ΔΔCT^ values.

### 4.9. Statistical Analysis

All data are expressed as the mean ± SD. One-way ANOVA with post-hoc Tukey HSD Test was used to compare pain (damaged limb weight distribution ratio), gene expression, histology, and gross morphology between groups. Differences were considered significant for *p*-values < 0.05. We used One-way ANOVA with post-hoc Tukey HSD Test and added a description on statistical service in https://astatsa.com/ (accessed on 18 April 2019).

## 5. Conclusions

In this study, we investigated the effects of lenvatinib on synovitis, osteophyte formation, and cartilage degradation in a rabbit OA model induced by ACLT. Oral administration of lenvatinib inhibited synovitis, osteophyte formation, and cartilage degeneration and reduced pain in a rabbit ACLT model. Lenvatinib is an oral VEGF inhibitor that is easier to administer than other VEGF inhibitors and may have potential as a treatment of posttraumatic OA.

## Figures and Tables

**Table 1:** Primers used for real-time polymerase chain reaction.

Target Gene	Primer Sequence: Forward	Primer Sequence: Reverse
*GAPDH*	5′-AAGGTCGGAGTGAACGGATT-3′	5′-TGGCGACAACATCCACTTTG-3′
*MMP3*	5′-ACACCGGATCTGCCAAGAGA-3′	5′-CTGGAGAACGTGAGTGGAGTCA-3′
*MMP13*	5′-GATGCCATTACCAGTCTCC-3′	5′-GCTGTATTCAAACTGTATGG-3′
*VEGFA*	5′-TGCCCACCGAGGAGTTCA-3′	5′-GGCCCTGGTGAGGTTTGAT-3′
*ADAMTS-5*	5′-GACAAGAGCCTGGAGGTGAG-3′	5′-AGGCATCGATACTGGTGAGG-3′

## Data Availability

The data presented in this study are available on request from the corresponding author. The data are not publicly available due to regulation of our facility.
